# Abbreviated gadoxetic acid–enhanced MRI versus ultrasonography for HCC surveillance in high-risk patients: A randomized trial protocol

**DOI:** 10.1097/HC9.0000000000000839

**Published:** 2025-12-01

**Authors:** Hyo Jung Park, Jonggi Choi, Dong Wook Kim, Sang Hyun Choi, Won-Mook Choi, Sung Won Chung, Danbi Lee, Ju Hyun Shim, Han Chu Lee, Young-Suk Lim, Min-Ju Kim, Amit G. Singal, Seong Ho Park, So Yeon Kim

**Affiliations:** 1 Department of Radiology and Research Institute of Radiology, Asan Medical Center, University of Ulsan College of Medicine, Seoul, Republic of Korea; 2 Department of Gastroenterology, Liver Cancer Center, Asan Medical Center, University of Ulsan College of Medicine, Seoul, Republic of Korea; 3 Department of Clinical Epidemiology and Biostatistics, Asan Medical Center, University of Ulsan College of Medicine, Seoul, Republic of Korea; 4 Department of Internal Medicine, University of Texas Southwestern Medical Center, Dallas, Texas, USA

**Keywords:** early detection of cancer, liver cirrhosis, liver neoplasms, magnetic resonance imaging, ultrasound imaging

## Abstract

**Background:**

Although ultrasonography (US) is widely recommended for HCC surveillance, its limited sensitivity for early-stage HCC remains a concern. Gadoxetic acid–enhanced abbreviated MRI (HBP-AMRI) has demonstrated high diagnostic performance; however, its role in routine surveillance settings remains uncertain due to limited prospective comparative evidence. The AMRIUS study (Abbreviated MRI using gadoxetic acid versus Ultrasonography for Surveillance of early-stage HCC in high-risk patients) aims to prospectively compare the effectiveness of HBP-AMRI and US for HCC surveillance in high-risk patients with cirrhosis.

**Methods:**

AMRIUS is a randomized controlled trial (RCT) enrolling 806 high-risk patients with cirrhosis, randomly assigned (1:1) to undergo either biannual HBP-AMRI or US for 2 surveillance rounds. The primary endpoint is the detection rate of early-stage HCC [Barcelona Clinic Liver Cancer (BCLC) stage 0 or A]. Secondary endpoints include false referral rate of BCLC 0 or A HCC, detection and false referral rates for BCLC stage 0 and all-stage HCC, BCLC stage distribution at initial HCC diagnosis, and the rate of curative treatment. Structured imaging protocols and quality assessments will be implemented.

**Discussion:**

AMRIUS is the first RCT designed to provide high-level evidence comparing HBP-AMRI and US for HCC surveillance. Its findings are expected to inform future guidelines and support risk-adapted strategies that prioritize early detection and curative treatment eligibility, particularly for patients likely to benefit from high-sensitivity imaging.

**Trial Registration:**

Registered at Clinical Research Information Service on May 22, 2022 (KCT0007417) and ClinicalTrials.gov on March 9, 2024 (NCT06312826). Participant recruitment began on August 26, 2022. Follow-up is ongoing.

## INTRODUCTION

HCC is the sixth most common cancer and the third leading cause of cancer-related deaths worldwide.^1^ To improve patient outcomes, regular surveillance aimed at detecting early-stage HCC is recommended in at-risk populations, as early detection allows for timely curative treatment. Ultrasonography (US) is recommended as the primary surveillance modality in major clinical guidelines^2^,^3^,^4^,^5^; however, it has limited sensitivity for detecting early-stage HCC in high-risk individuals.^6^,^7^,^8^,^9^ A meta-analysis reported that the pooled sensitivity of US alone for detecting early-stage HCC in patients with cirrhosis was 47%,^6^ and prospective have shown a wide range of sensitivities, from 25.6% to 62.5%.^10^,^11^,^12^,^13^,^14^ Moreover, US performance varies considerably depending on factors such as imaging protocols, equipment quality, patient body habitus, liver morphology, and the operator’s level of expertise.^15^,^16^

To overcome these limitations, alternative imaging approaches are being explored for HCC surveillance in high-risk populations.^17^ Among these, gadoxetic acid–enhanced MRI has shown excellent performance in detecting HCC,^12^ primarily through hepatobiliary phase (HBP) imaging acquired 15–20 minutes after the intravenous injection of gadoxetic acid. This phase enhances lesion-to-liver contrast by exploiting hepatocyte-specific uptake of gadoxetic acid, facilitating early detection of small HCCs.^17^,^18^,^19^ However, the widespread application of full MRI for surveillance is limited by high cost, long scan time, and resource constraints. Abbreviated MRI using HBP imaging (HBP-AMRI) has demonstrated excellent diagnostic performance while reducing scan time by omitting dynamic contrast-enhanced sequences. Retrospective studies have consistently reported high sensitivity of HBP-AMRI for detecting HCC, ranging from 82.6% to 92.0%, and specificity ranging from 91.0% to 93.2%.^20^,^21^,^22^,^23^,^24^,^25^ However, these findings may be affected by selection bias, as the studies were primarily conducted in retrospective diagnostic contexts with a high HCC prevalence (14.9%–35.6%) and typically employed single-session AMRI rather than repeated imaging over time, which may limit their applicability to true surveillance settings. In addition, several studies used MRI as the reference standard,^21^,^22^,^23^,^25^ with both the simulated AMRI and the reference MRI derived from the same imaging session, introducing a lack of independence between the index test and the reference standard.

While HBP-AMRI is promising, robust comparative evidence against US in a surveillance setting is lacking. Prior studies employing single-arm or intraindividual designs^12^,^14^,^24^ may have underestimated the performance of US, as surveillance was often discontinued when MRI detected HCC earlier than US. This early termination limits direct temporal comparison between the 2 modalities. To address this limitation and provide high-quality evidence on their comparative effectiveness in HCC surveillance, we initiated a prospective, randomized controlled trial (RCT) to compare biannual US and biannual HBP-AMRI in high-risk patients with cirrhosis.

## METHODS

### Study design

The current trial [Abbreviated MRI using gadoxetic acid versus Ultrasonography for Surveillance of early-stage HCC in patients at high risk (AMRIUS)] is a prospective, single-center, open-label RCT designed to compare the efficacy of biannual HBP-AMRI and biannual US for the surveillance of HCC in high-risk patients with cirrhosis. The trial is being conducted at Asan Medical Center, a tertiary academic hospital in the Republic of Korea, which has 2432 beds and performs over 3000 liver imaging surveillance examinations annually, ensuring procedural consistency and technical expertise.

Participants meeting the eligibility criteria are randomly assigned in a 1:1 ratio to undergo either HBP-AMRI or US every 6 months during 2 consecutive surveillance rounds. The trial has been registered at ClinicalTrials.gov (NCT06312826) and in the Clinical Research Information Service of South Korea (KCT0007417). Participant recruitment began on August 26, 2022. Follow-up for primary and secondary endpoints is ongoing.

### Ethics approval and informed consent

This study was approved by the Institutional Review Board (Asan Medical Center; IRB number: 2022-0661). The trial is being conducted in accordance with the Declaration of Helsinki and Good Clinical Practice guidelines. Any modifications to the protocol will be implemented only after Institutional Review Board approval and notification to all participating investigators. Written informed consent will be obtained from all participating patients.

### Eligibility criteria

#### Inclusion criteria

1. Adults aged ≥20 years.
2. Diagnosis of liver cirrhosis based on at least 1 of the following:
  Histological confirmation via liver biopsy, or
  Radiologic evidence of cirrhosis and portal hypertension, defined as typical imaging features of liver cirrhosis on radiologic examination with liver stiffness ≥12 kPa on transient elastography or the presence of esophageal or gastric varices.
3. High risk for HCC (estimated annual risk >5%), defined as a risk index ≥2.33:
  Risk index=1.41 (if age ≥50 y) + 1.65 (if prothrombin activity ≤75%) + 0.92 (if platelet count ≤100 × 10^3^/μL) + 0.74 (if positive for anti-HCV or HBsAg).^26^
4. Eastern Cooperative Oncology Group performance status 0–2.
5. No history or current evidence of HCC within 6 months prior to enrollment.
6. Willing and able to provide written informed consent and comply with study procedures.

#### Exclusion criteria

1. Presence of an active or suspected cancer, or a history of treated malignancy with a risk of recurrence ≥20% within 2 years.
2. Significant comorbidity with an expected survival of <3 years.
3. Estimated glomerular filtration rate <30 mL/min/1.73 m^2^.
4. Child–Pugh class C.
5. Conditions precluding application of the Liver Imaging Reporting and Data System (LI-RADS) (eg, Budd–Chiari syndrome or other hepatic vascular disorders).
6. Contraindications to MRI (eg, pacemakers, severe claustrophobia, pregnancy).
7. Any condition deemed by the investigators to interfere with study participation or protocol adherence.

### Sample size

The sample size was calculated based on a 2-sided test for comparing 2 independent proportions, with the primary endpoint being the detection rate of early-stage HCC. Based on prior prospective studies in real-world surveillance settings,^12^,^13^,^14^ we assumed a cumulative prevalence of very early or early-stage HCC of 8% during the study period. The sensitivity of HBP-AMRI was estimated at 90%, and that of US at 35%, reflecting the range observed in surveillance cohorts of patients with cirrhosis.^12^,^13^,^14^ Accordingly, we assumed detection rates of 7.2% for HBP-AMRI and 2.8% for US. To detect a statistically significant difference in detection rates between the 2 modalities, with 80% power and a 2-sided alpha of 0.05, and accounting for an anticipated 5% dropout rate, the total required sample size is 806 participants (403 per arm).

### Enrollment and allocation

Participants were randomly assigned in a 1:1 ratio to either the HBP-AMRI arm or the US arm using a permuted block randomization method with a random block size of 4. Allocation concealment was ensured through the use of a sequestered code, accessed only after informed consent was obtained. The study is open-label, as both participants and investigators were aware of the surveillance assignment due to the visible procedural differences between US and HBP-AMRI.

### Participant flow

A trained research coordinator introduced the trial to potentially eligible patients during outpatient clinic visits or scheduled assessments. Patients who met all eligibility criteria received comprehensive verbal and written information about the study’s objectives, procedures, risks, and potential benefits. Adequate time was provided for consideration, and informed discussions with investigators were encouraged. Written informed consent was obtained from those who agreed to participate.

**FIGURE 1:**
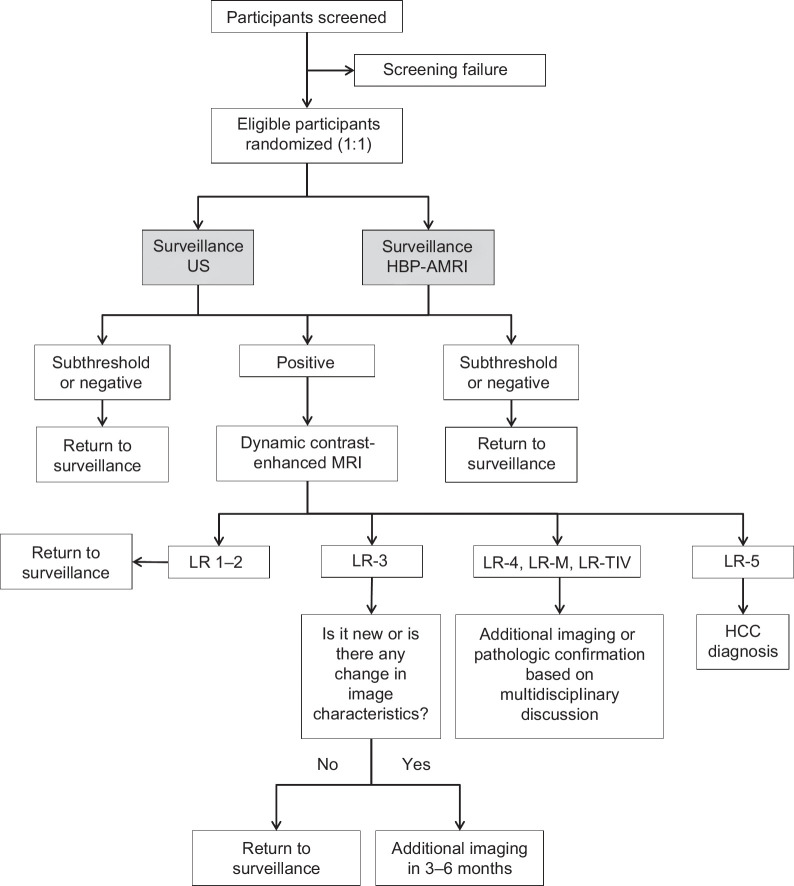
Participant flow. Abbreviations: HBP-AMRI, hepatobiliary phase-abbreviated magnetic resonance imaging; LR, Liver Imaging Reporting and Data System category; US, ultrasonography.

Upon enrollment, participants were randomly assigned in a 1:1 ratio to undergo 2 consecutive rounds of HCC surveillance using either biannual US or biannual HBP-AMRI at 6-month intervals (with a variation of ±1 mo allowed) (Figure 1). The first surveillance test is scheduled for ~6 months after the participant’s last imaging study, confirming the absence of hepatic malignancy.

If a lesion is detected and considered potentially malignant based on predefined radiologic criteria (see *Imaging Evaluation* section below), a confirmatory imaging test will be conducted. Participants with no suspicious findings will proceed to the second surveillance round at the scheduled interval. Following completion of the second surveillance round, all participants will enter a 6-month observation period, during which delayed HCC diagnosis and clinical outcomes will be assessed. If a “positive” lesion is ultimately determined to be a false-positive following the confirmatory test, the participant will return to their assigned surveillance schedule.

Dropout will be defined as: (1) withdrawal of consent or determination by the investigators that the participant is unable to adhere to the protocol; (2) liver transplantation without prior HCC diagnosis, (3) diagnosis of extrahepatic malignancy during the study period, or (4) failure to complete one or more consecutive scheduled surveillance visits. To minimize dropouts, participants will receive up to 2 reminders per visit (3 d and 1 d in advance) via phone call or text message. In addition, staff will offer flexible scheduling support and follow-up if participants miss or need to reschedule an appointment.

Peripheral blood samples will be collected at the screening visit and at each surveillance visit for all enrolled participants following approved protocols. Serum levels of alpha-fetoprotein and protein induced by vitamin K absence-II will be measured at each visit. Stored samples may be used in future ancillary studies. Written informed consent will be obtained from all enrolled participants for specimen collection and future use.

### Ultrasonography

Abdominal US will be performed using a commercially available scanner (Aplio i800, Canon Medical Systems, Otawara, Japan) equipped with a 5-MHz curved-array transducer. All surveillance US examinations will be conducted at Asan Medical Center by board-certified abdominal radiologists. US will be performed following a standardized acquisition protocol recommended by US LI-RADS v2017^27^ and LI-RADS US Surveillance v2024.^28^ Images will be digitally archived for quality assurance and secondary review.

### Abbreviated MRI (HBP-AMRI)

HBP-AMRI will be conducted using 3.0-T scanners available at the study site, including Achieva and Ingenia (Philips Healthcare, Best, the Netherlands), Skyra and Vida (Siemens Healthcare, Erlangen, Germany), and Architect and Premier (GE Healthcare, Milwaukee, WI, USA). All participants will receive a gadoxetic acid injection (Gd-EOB-DTPA, Primovist, Bayer Schering Pharma) at a dose of 0.025 mmol/kg, followed by a 20-mL saline flush in the preparation room. After a 15–20-minute delay, participants will move into the MRI suite for scanning. The protocol will include the following axial sequences: (1) heavily T2-weighted imaging (repetition time, 1100 ms; echo time, 150 ms; slice thickness, 5 mm); (2) respiratory-triggered or free-breathing diffusion-weighted imaging with b-values of 50, 500, and 900 s/mm^2^ (repetition time, 2000 ms; echo time, 54–56 ms; slice thickness, 5 mm) with ADC map reconstruction; and (3) fat-suppressed 3D gradient-echo T1-weighted imaging during HBP using manufacturer-specific sequences [eg, VIBE (Siemens), LAVA (GE), or THRIVE (Philips)] (repetition time, 3.4 ms; echo time, 1.3 ms; slice thickness, 3 mm). Scan time for HBP-AMRI is expected to be under 15 minutes, depending on equipment and patient cooperation.

### Imaging evaluation

All surveillance imaging studies will be interpreted by board-certified abdominal radiologists using a standardized 3-point scale reflecting the likelihood of HCC: positive, subthreshold, and negative (Table 1). Participants with a “positive” result on either modality will undergo confirmatory testing, while those with a “subthreshold” or “negative” result will proceed to the next scheduled surveillance in 6 months.

**TABLE 1:** Lesion categorization on HBP-AMRI and US

Category	HBP-AMRI	US
	Definition	Definition
Positive	▪ A new solid nodule ≥10 mm is not definitely benign^a^ ▪ Changes in imaging characteristics^b^ or threshold growth^c^ of a previous subthreshold observation ▪ New thrombus in the vein	• A new solid nodule or parenchymal distortion ≥10 mm distinct from the background liver • Threshold growth^c^ of previous subthreshold observation • New thrombus in the vein
Subthreshold	▪ Solid nodule <10 mm, not definitely benign^a^ ▪ Previous positive observation confirmed as a false positive	• Solid nodule or parenchymal distortion <10 mm • Previous positive observation confirmed as a false positive
Negative	▪ No observation ▪ Only definitely benign observation^d^	• No observation • Only definitely benign observation^d^

^a^ Mild to moderate hyperintensity on T2-weighted imaging, diffusion restriction, or hypointensity on hepatobiliary-phase imaging.

^b^ Any new appearance of not definitely benign characteristics.

^c^ Size increase by ≥50% in ≤6 months.

^d^ Cyst, hemangioma, or other previously characterized benign lesion.

Abbreviations: HBP-AMRI, hepatobiliary phase-abbreviated magnetic resonance imaging; US, ultrasonography.

For HBP-AMRI, a “positive” result is defined as the presence of a new solid nodule ≥10 mm that does not demonstrate definitely benign characteristics (mild to moderate hyperintensity on T2-weighted imaging, diffusion restriction on diffusion-weighted imaging, or hypointensity on HBP imaging), changes in imaging characteristics or threshold growth of a previous subthreshold observation, or development of new thrombus in vein (TIV). Benign entities such as cysts, hemangiomas, and previously characterized benign lesions are not considered positive, regardless of size.

For US, classification follows the criteria of US LI-RADS v2017^27^ and LI-RADS US Surveillance v2024.^28^ A “positive” result is defined as a new solid nodule or parenchymal distortion ≥10 mm that is distinct from the background liver, threshold growth of a previous subthreshold observation, or new TIV. Cysts, hemangiomas, or previously characterized benign lesions are not considered positive findings regardless of size. For both HBP-AMRI and US, image quality will be classified as A (no or minimal limitation), B (moderate limitation), or C (severe limitation), as previously described^19^,^28^ (Table 2).

**TABLE 2:** Classification of image quality

Score	HBP-AMRI	US
A (no or minimal limitation)	▪ Liver signal on HBP is considerably higher than that of the vessels ▪ Liver texture: homogeneous or mildly heterogeneous	▪ Liver is homogeneous or minimally heterogeneous ▪ Minimal beam attenuation or shadowing ▪ Liver visualized in near entirety
B (moderate limitation)	▪ Liver signal on HBP is only slightly higher than that of the vessels ▪ Liver texture is moderately heterogeneous	▪ Liver moderately heterogeneous ▪ Moderate beam attenuation or shadowing ▪ Some portion of the liver or diaphragm is not visualized
C (severe limitation)	▪ Liver signal on HBP is the same or lower than that of the vessels ▪ Liver texture is severely heterogeneous ▪ Severe artifact	▪ Liver severely heterogeneous ▪ Severe beam attenuation or shadowing ▪ The majority (>50%) of the liver or diaphragm is not visualized

Abbreviations: HBP, hepatobiliary phase; HBP-AMRI, hepatobiliary phase-abbreviated magnetic resonance imaging; US, ultrasonography.

### Reference standards

The reference standard for final diagnosis will begin with radiologic confirmation using a complete dynamic contrast-enhanced MRI with an extracellular contrast agent (Dotarem; gadoterate meglumine; Guerbet, Roissy, France), interpreted according to CT/MRI LI-RADS v2018.^29^ Lesions classified as LR-5 will be considered diagnostic of HCC. For lesions classified as LR-4, LR-M, or LR-TIV, additional imaging evaluation—including liver dynamic CT, dynamic contrast-enhanced MRI with gadoxetic acid, or contrast-enhanced US or histologic confirmation via US-guided biopsy—will be performed based on multidisciplinary discussion.

For lesions classified as LR-3, if the lesion is pre-existing without interval change, the participant will return to the study and undergo subsequent surveillance testing. If the LR-3 lesion is newly identified or shows any change in image characteristics (ie, new appearance of mild to moderate T2 hyperintensity, diffusion restriction, or hypointensity on HBP imaging), follow-up imaging will be performed within 3–6 months. Participants with the lesion classified as LR-1 or LR-2 will return to the study and undergo subsequent surveillance testing.

### Outcomes

The primary endpoint of this trial is the detection rate of early-stage HCC, defined as Barcelona Clinic Liver Cancer (BCLC) stage 0 or A.^30^ Detection rate is defined as the number of patients whose BCLC 0 or A HCC is detected using a given surveillance modality, divided by the total number of patients under surveillance with that modality, expressed as a percentage. Secondary endpoints include the false referral rate of BCLC 0 or A HCC, detection and false referral rates of BCLC stage 0 HCC, detection and false referral rates of all-stage HCC, distribution of BCLC stages at initial HCC diagnosis, and the rate at which curative treatment (surgical resection and local ablative therapies including radiofrequency ablation, microwave ablation, and cryoablation) is received. False referral rate is defined on a per-exam basis, calculated as the number of surveillance tests interpreted as positive but subsequently confirmed as non-malignant on confirmatory examination, divided by the total number of surveillance tests performed in that modality arm, expressed as a percentage.

### Data collection and management

All study data are collected and managed using an electronic case report form hosted on a secure, web-based platform (https://amrius.amc.crf.kr, Procuratio, Seoul, Korea). Investigators and trained research coordinators enter relevant clinical, imaging, and laboratory data into the electronic case report form at each surveillance visit. Upon enrollment, each participant was assigned a unique study identification number, which is used throughout the study to maintain data anonymity. A designated data manager performs regular quality checks for completeness and accuracy. All data modifications are recorded and logged for audit trail purposes. Personal identifiers are stored separately from study data and protected using coding procedures. Only authorized personnel (investigators, coordinators, data managers) have access to the full dataset.

### Statistical analysis

Detection rates and the rate of curative treatment will be compared using the Fisher exact test. BCLC stage distribution will be compared using the chi-square test. False referral rates will be compared using Fisher exact test or Poisson regression analysis. Time to tumor detection will be analyzed using Kaplan–Meier methods and compared using the log-rank test. Associations between liver function as assessed by Child–Pugh (ie, Child–Pugh A or B), and surveillance image quality, as well as between image quality and detection or false referral rates, will be examined using chi-square or Fisher exact tests as appropriate. If appropriate, multivariable logistic regression analyses will be used to determine the independent associations between variables. All tests will be 2-sided, with *p*-values <0.05 considered statistically significant. Statistical analyses will be conducted using SAS version 9.2 (SAS Institute Inc., Cary, NC, USA).

## DISCUSSION

The aim of the AMRIUS trial is to determine whether surveillance using HBP-AMRI with gadoxetic acid improves early HCC detection performance in high-risk patients with cirrhosis compared with conventional US. We are employing a 2-arm randomized design to enable a direct comparison of the 2 imaging modalities while minimizing bias, thereby providing robust evidence to evaluate the potential superiority of HBP-AMRI over US.

Early detection of HCC plays a pivotal role in surveillance, as curative treatments such as surgical resection and ablation are feasible when HCCs are identified at an early stage, typically BCLC stage 0 or A. To align with this clinically meaningful goal, we defined the primary endpoint as the detection rate of BCLC stage 0 or A HCC. In addition, the false referral rate, included as a key second endpoint, captures the broader impact of surveillance by reflecting unnecessary diagnostic procedures, patient anxiety, and healthcare resource consumption.^31^,^32^,^33^ Furthermore, by investigating the initial stage distribution of detected HCCs, we intend to assess the extent to which each surveillance modality enables detection at a stage amenable to curative treatment.

Among at-risk individuals, the likelihood of HCC varies according to underlying etiology (eg, viral hepatitis, alcohol-associated liver disease, or metabolic dysfunction–associated steatotic liver disease), degree of liver fibrosis, and other clinical parameters. As such, a uniform surveillance strategy may not be optimal, and recent guidelines and expert recommendations increasingly support risk-stratified surveillance tailored to individual patient profiles.^33^ By enrolling patients with a quantitatively defined high risk for HCC (estimated annual risk >5%), the AMRIUS trial is positioned to contribute evidence toward this evolving strategy. Its findings may help identify subgroups that derive the greatest benefit from high-sensitivity modalities such as HBP-AMRI and also provide evidence on the most suitable surveillance modality for these populations, thereby supporting the future development of personalized surveillance strategies.

In contrast to prior intraindividual comparison studies of AMRI and US^12^,^14^,^24^ and ongoing prospective studies of HBP-AMRI and US (NCT04539717, NCT04288323), the AMRIUS trial is a randomized study designed to avoid bias caused by the higher sensitivity of MRI, which can lead to earlier detection of lesions that might have otherwise been detected later by US, thereby underestimating the performance of US. The recently published MIRACLE trial^34^ compared non-contrast AMRI with US in a randomized setting, reporting that the non-contrast MRI group detected tumors at earlier BCLC stages (*p*=0.01) and had a lower false-positive referral rate (*p*<0.001). However, the study failed to find a difference in early-stage detection rates and included an unstratified population with cirrhosis, limiting its applicability to risk-stratified surveillance. AMRIUS specifically targets high-risk patients and employs HBP imaging with gadoxetic acid, providing hepatocyte-specific contrast enhancement and excellent sensitivity for early-stage HCC. By integrating risk stratification with a contrast-enhanced abbreviated protocol, AMRIUS seeks to provide more robust evidence for optimizing surveillance strategies. Comparison of results from AMRIUS with those of other ongoing RCTs comparing different types of AMRI and US—such as non-contrast AMRI versus US (NCT05095714, NCT05429190) and dynamic contrast-enhanced AMRI versus US (NCT05486572)—is also anticipated.

Another important strength of the AMRIUS study lies in its use of an independent reference standard, employing an extracellular contrast agent to ensure an unbiased assessment of AMRI performance. Among the retrospective studies assessing the potential of AMRI as a surveillance test,^20^,^21^,^22^,^23^,^24^,^25^ several used simulated AMRI constructed from selected sequences of the same complete contrast-enhanced MRI that also served as the reference standard.^21^,^22^,^23^,^25^ In these studies, the diagnostic performance of AMRI was evaluated using the same MRI scans from which the abbreviated protocols were derived, resulting in a lack of independence between the index test and the reference standard. This methodological limitation may have inflated the reported diagnostic accuracy of AMRI and limited the applicability of their findings to real-world surveillance settings, where independent confirmatory imaging is required. The AMRIUS study incorporates independent confirmatory MRI using an extracellular contrast agent, thus avoiding this limitation and enabling an unbiased evaluation of AMRI performance in a true surveillance context.

Another strength of the AMRIUS trial is the integration of structured reporting and image quality assessment, which have been underexplored in prior prospective studies.^14^,^34^ To ensure consistency and clarity, standardized interpretation templates will be used for both HBP-AMRI and US, with the US template will follow the LI-RADS framework,^27^,^28^ facilitating uniform assessments and effective communication with referring physicians. In addition, the reference standard, complete dynamic contrast-enhanced MRI, will also be interpreted in a standardized manner according to CT/MRI LI-RADS v2018.^29^ Structured image quality evaluation will also be implemented to assess modality appropriateness and support individualized surveillance planning, such as timely switching in cases of suboptimal image quality. Given that impaired liver function can reduce MRI quality and compromise its sensitivity, capturing HBP-AMRI quality will generate essential data for optimizing MRI-based surveillance in clinical practice.

While AMRIUS focuses on imaging-based surveillance, it is also important to consider the broader landscape of evolving surveillance strategies. Ongoing parallel efforts are underway to validate serum biomarkers, including methylated DNA panels (ALTUS [NCT05064553] and the CliMB study [NCT03694600]) and the GALAD score as studied in the TRACER trial [NCT06084234].^35^ These developments highlight the potential for more personalized surveillance frameworks that integrate individual patient risk, imaging, and biomarker data.

This study is limited by the fact that it is single-center and conducted at a large tertiary referral hospital with well-equipped imaging facilities and highly experienced professionals, particularly regarding surveillance MRI. While this setting ensures high-quality image acquisition and interpretation, it may limit the generalizability of the findings to other institutions with different resources and infrastructure.

In conclusion, the AMRIUS trial is the first RCT to compare the effectiveness of HBP-AMRI and US in the surveillance of HCC in high-risk patients with cirrhosis. We expect that the findings from this trial will provide high-quality evidence supporting the efficacy of HBP-AMRI as a surveillance tool for HCC in this patient population.

## Data Availability

Data collection is ongoing. Data sharing is not applicable to this article, as no datasets were generated or analyzed during the current study. So Yeon Kim conceptualized this study. All authors (Hyo Jung Park, Jonggi Choi, Dong Wook Kim, Sang Hyun Choi, Sung Won Chung, Won-Mook Choi, Danbi Lee, Ju Hyun Shim, Han Chu Lee, Young-Suk Lim, Min-Ju Kim, Amit G. Singal, and So Yeon Kim) participated in the study design. Jonggi Choi, Sung Won Chung, Won-Mook Choi, Danbi Lee, Ju Hyun Shim, Han Chu Lee, and Young-Suk Lim played major roles in patient enrollment. Min-Ju Kim provided professional statistical assistance. Hyo Jung Park, Jonggi Choi, Dong Wook Kim, Sang Hyun Choi, Young-Suk Lim, and So Yeon Kim established the protocols for surveillance MRI and US. Hyo Jung Park, Dong Wook Kim, Sang Hyun Choi, and So Yeon Kim performed the interpretation of the surveillance MRI and US. Hyo Jung Park was a major contributor to writing the manuscript. All authors read and approved the final manuscript. This study was supported by a grant from Bayer Korea, Co. Ltd. The funder has no role in study design, data collection and analysis, decision to publish, or preparation of the manuscript.
